# Our Blind Spots in the Fight Against Health Systems Corruption: Comment on "We Need to Talk About Corruption in Health Systems"

**DOI:** 10.15171/ijhpm.2019.81

**Published:** 2019-10-14

**Authors:** Reinhard Huss

**Affiliations:** Nuffield Centre for International Health and Development (LIHS), University of Leeds, Leeds, UK.

**Keywords:** Systemic Corruption, Health Sector, Leadership, Finance, Research

## Abstract

The health sector often appears prominent in surveys of perceived corruption, because citizens experience the symptoms of systemic corruption most distressingly during their interaction with frontline health workers. However, the underlying drivers of systemic corruption in society may be located in other social systems with the health system demonstrating the symptoms but not the path how to exit the situation. We need to understand the mechanisms of systemic corruption including the role of corrupt national and international leaders, the role of transnational corporations and international financial flows. We require a corruption definition which goes beyond an exclusive focus on the corrupt individual and considers social systems and organisations facilitating corruption. Finally there is an urgent need to address the serious lack of funding and research in the area of systemic corruption, because it undermines the achievement of the Sustainable Development Goals (SDGs) in many low income countries with the most deprived populations.

## Introduction

Recently your journal has published an editorial “We Need to Talk About Corruption in Health Systems”^1^ and a commentary “Opening the Policy Window to Mobilize Action Against Corruption in the Health Sector.”^2^ Both articles have raised important issues of health sector and health systems corruption. The health sector is vulnerable to corruption for several reasons which have been discussed elsewhere.^3^ In a society with widespread or systemic corruption citizens are likely to experience the symptoms of corruption in their interaction with frontline health workers. However, similar to the treatment of fever in Malaria which does not cure the disease, a focus on the corrupt symptoms in the health sector does not address the underlying societal causes of widespread corruption. These issues are not or only briefly mentioned in the editorial and the commentary. The challenges include the inadequate definition of corruption which needs to consider organisational and systemic corruption outside individual control and beyond the health sector, corruption in the political and administrative leadership of a country, the corrupt behaviour of international corporations and political leaders as successful role models, global financial corruption facilitating national corruption and the lack of research about the underlying drivers of systemic corruption.

## Corruption Definition

The definition of corruption determines what we perceive as the problem and how it will be tackled. The change of definition from the misuse of public office for private gain^4^ to “abuse of entrusted power for private gain” (pxvii),^5^ has addressed the misleading concept that privatisation may get rid of corruption. However, both definitions do not clearly take account of organised and systemic corruption which is part of the original meaning of the term corruption as the decay and putrefaction of a system. In Table 1 the author compares individual, organisational, and systemic corruption. While there are no universally agreed definitions of organisational and systemic corruption,^6^ certain characteristics appear to be common.

**Table 1:** Comparison of Individual, Organisational and, Systemic Corruption

**Level**	**Individual**	**Organisational**	**Systemic**
Definition	Abuse of entrusted power for private gain^5^	Abuse of entrusted power at organisational level for private gain	Deliberate betrayal of public trust and the undermining of the public good for private gain^7^
Main actors	Individuals	Private companies and government of state organisation	Individuals, companies, governments
Description	Common corrupt practices which are the focus of corruption research and intervention. These are also part of organisational and institutional corruption.	Unethical or illegal and opaque contract arrangements which are not Following agreed good governance procedures between private companies and government. Corruption has become a way to finance politicians and political parties.	Several or most societal sectors function on the basis of corrupt practices. Corruption has been normalized and becomes the ‘way of life.’
Main mechanism	Corrupt actors use deficiencies and inconsistencies in management procedures and the rule of law.	Corrupt actors use the contacts to high-ranking government officials and their interest in corrupt deals.	Rule of power has replaced the rule of law.
Beneficiaries	Individuals directly involved in corrupt practices and their associates.	Individuals involved in contract procedures but also government and company leadership, senior management and potentially owners of company without direct involvement in corrupt action. Disguise of plausible denial because of lack of transparency and written evidence.	Individuals and organisations at the top of the power hierarchy in society benefit to a large extent while many citizens are involved to a certain extent in order to survive in society. Poor citizens at the bottom of the hierarchy suffer most from systemic corruption.
Example health sector	Frontline health professionals demand bribes from patients in exchange for treatment.	Order of pharmaceutical goods includes a kickback for senior government officials. Kickbacks are paid into foreign bank accounts where financial flows and owners are protected from public scrutiny.	Citizens or health professionals who complain about corrupt practices to an ombudsperson or anti-corruption agency are threatened and harmed.

The main role of the state to protect the public good can be undermined when powerful societal actors capture the state or some of its organs and weaken the universal functions of the state for private organisational gain.^7^ Typical examples from the health sector are the deliberate sale and dissemination of counterfeit and sub-standard medicines and diagnostics or the purchase of pharmaceutical goods at an excessive price (Table 1). Organisational tax evasion and avoidance are practices that undermine public goods such as universal health services and ethical standards in society.

However, there is an important difference between tax evasion and avoidance. The former is illegal, while the latter is legal and some citizens, especially with a neoliberal ideology, for whom private gains take precedence over the common good, may describe it as an ethical practice. Illegal corruption is easy to define as a person with entrusted power who uses the position for private gains. The corrupt practices vary and include the demand of gifts or cash for public services. These are clearly defined as illegal in most state jurisdictions. It is more difficult to agree whether a legal practice is unethical and corrupt. Should tax avoidance through transfer pricing of a company and tolerated by the tax authority be defined as a legal but unethical and corrupt practice, because it undermines the capacity of the state to provide universal public services? The answer is yes, in case we apply Lessig’s definition of institutional corruption^8^: “Institutional corruption is manifest when there is a systemic and strategic influence which is legal, or even currently ethical, that undermines the institution’s effectiveness by diverting it from its purpose or weakening its ability to achieve its purpose, including, to the extent relevant to its purpose, weakening either the public’s trust in that institution or the institution’s inherent trustworthiness.” The worst case is systemic corruption when several societal institutions such as police, judiciary, tax authority and others are undermined by corrupt practices.

## Systemic Corruption and the Health System

With systemic corruption corrupt behaviour becomes an integral part of the economic, social and political systems in a society.^9,10^ Some have described it as endemic or institutionalised corruption. Citizens have to learn how to deal with corrupt officials. It becomes normal practice in transactions between public servants and individuals or businesses. There are strong incentives for everybody to comply with this illegitimate system.

Most sectors are affected and interventions need to target at least several key or all sectors. Interventions with an exclusive focus on the health sector are unlikely to succeed, because the state and its different organs such as legislature, executive, judiciary, police and anti-corruption agencies are seriously affected by corruption.^7^

There are no specific and agreed indicators of systemic corruption. However, high levels of public sector corruption as determined by the corruption perception index of Transparency International^11^ are likely to point to systemic corruption. The combined estimated performance of 33 health-related Sustainable Development Goal (SDG) indicators based on the Global Burden of Disease study^12^ provides some indication of societal achievement related to health. The author selected the bottom 20 out of 188 countries in terms of health achievement and compared their ranking in the corruption perception index of the same year (Table 1). Many of the worst performers in health are associated with a high level of perceived corruption.

There are windows of opportunity to redress societies, reduce and overcome systemic corruption. This seems to have happened to some extent in Rwanda after the genocide in 1994^13^ and in Singapore in 1960 after the enactment of the Prevention of Corruption Act.^14^ In these two countries it was mainly due to national drivers of change.

Unfortunately most of the time the international community is willing to overlook even blatant cases of political and systemic corruption. This was demonstrated by the recent presidential election in the Democratic Republic of the Congo in 2018/2019.^15^ One consequence is the deep mistrust of citizens in the government which has a serious impact on the control of the actual Ebola epidemic in the country.^16^ It is also reflected in the poor position of the country in terms of perceived corruption and health achievement (Table 2). Another typical example is Angola with enormous petroleum wealth but poor health indicators and high levels of perceived corruption. Instead the country has produced Africa’s richest woman who also happens to be the daughter of the country’s former long-standing president.^17^

**Table 2:** Comparison of Positions Based on Health-Related SDG Indicators and Corruption Perception Index

**Country**	Position out of 188 Countries (**) ^12^	Position out of 168 Countries (*), (**) ^11^
Lesotho	169	061
Angola	170	163
Cameroon	171	130
Burkina Faso	172	076
Uganda	173	139
Ethiopia	174	102
Guinea	175	139
Guinea-Bissau	176	158
Mozambique	177	111
Madagascar	178	123
Sierra Leone	179	119
Afghanistan	180	166
Mali	181	095
Burundi	182	150
Democratic Republic of the Congo	183	147
Chad	184	147
Niger	185	098
South Sudan	186	163
Somalia	187	167
Central African Republic	188	145

Abbreviation: SDG, Sustainable Development Goal. Note: (*) Several countries can hold the same position in the index. The next lower country will receive the rank which equals the number of countries in the position above this country plus 1. (**) Lower rank indicates worse situation.

Societies with embedded systemic corruption are at high risk to slide into a vicious cycle of sustainable corruption. Leaders and corporate organisations at the top of the national and international power hierarchy profit from the situation and have little interest in radical change. Corrupt politicians and corporations attract others with similar interests and deter any promoters of good governance. An opaque international financial system which is not accountable to the public good of societies facilitates corrupt practices. An international governance system where unconditional national sovereignty is a supreme principle prevents collective international action. The next sections describe some of the factors facilitating systemic corruption.

## Corrupt Decision-Makers in Leading Government Positions

This issue is closely linked to systemic corruption. Many anti-corruption interventions fail, because international organisations wrongly assume that the government has the political will to fight against corruption. Persson et al^18^ “argue that part of an explanation to why anticorruption reforms in countries plagued by widespread corruption fail is that they are based on a theoretical mischaracterization of the problem of systemic corruption.…the analysis reveals that while contemporary anticorruption reforms are based on a conceptualization of corruption as a principal–agent problem, in thoroughly corrupt settings, corruption rather resembles a collective action problem.” The anticorruption reform wrongly assumes principled principals. In reality we are confronted with a breakdown of trust and reputation in society which leads to a failure to sanction corrupt behaviours and change societal rules. Ostrom^19^ defines it as a collective action problem of the second order.

## Behaviourof International Leaders and TransnationalCorporations

Your commentary mentions the seminal 1996 speech of World Bank President James Wolfensohn against the cancer of corruption. A big challenge is the consistency and credibility of the international discourse. His successor in the World Bank was Mr. Paul Wolfowitz. He had to resign from his position because of clear evidence of rule violations and attempts to weaken whistle-blower protection within the organisation.^20^

President Trump practices nepotism^21,22^ and appoints family members into White House positions. Private gains from these appointments may not be illegal but they are clearly unethical.^23^ Whether you classify it as corrupt behaviour may depend on the definition of corruption. Mr. Trump was the first major-party presidential nominee in more than 40 years who did not to release his tax returns, and he is the first president since the early 1970s to decline to release tax information, either through a summary or a full or partial return. This political and ethical standard setting of the President of the United States may be used to justify similar leadership behaviour in other countries.

Unfortunately the behaviour of transnational corporations often puts profits before the health of the public. The excess Nitrogen oxides emissions of Volkswagen cause premature deaths and enormous costs for the public health systems in European^24^ and other countries The same probably applies to other car manufacturers using similar manipulative ‘defeat devices’ to lower emissions during vehicle testing for regulatory purposes.

The classification of Glyphosate as probably carcinogenic to humans by a group of experts at the International Agency for Research on Cancer, a body of the World Health Organisation,^25^ has drawn attention to the fact how transnational corporations such as Monsanto attempt to influence and manipulate science in order to give priority to profit over public health. The key issue here is not the carcinogenicity of the substance, but how the power of a company is used to confuse the public and discredit scientists.^26^ Whether you classify these leadership and corporate behaviours as corrupt depends again on the definition of corruption which you want to apply.^7,9,10,21,27^ These cases affect public health and international ethics and leave health professionals and the public with the perception that the discourse of global corruption prevention and control may have a focus on the small rather than the big fish.

## Financing of Health Systems

The opaque international financial system which is not accountable to the public good at the national or international level facilitates corrupt practices. Illegal tax evasion and legal but unethical tax avoidance are major obstacles to adequate health system financing. The OXFAM report “Prescription for Poverty”^28^ states “four pharmaceutical corporations—Abbott, Johnson & Johnson, Merck, and Pfizer—systematically stash their profits in overseas tax havens. They appear to deprive developing countries of more than $100 million every year—money that is urgently needed to meet the health needs of people in these countries—while vastly overcharging for their products. And these corporations deploy massive influencing operations to rig the rules in their favour and give their damaging behaviour a veneer of legitimacy. Tax dodging, high prices and influence peddling by drug companies exacerbate the yawning gap between rich and poor, between men and women, and between advanced economies and developing ones.”

The Corporate Tax Haven Index^29^ explains how certain jurisdictions are used by transnational corporations to escape paying tax and how this erodes the tax revenues of countries around the world. Among the top ten tax havens are four British overseas territories or crown dependencies, two member states of the European Union and one other European state.

If we want to understand the mechanisms and chains of corruption, it is not sufficient to focus on the behaviour of frontline health workers. When working in the Central African Republic (bottom position in Table 2) from 1992 to 1998 government health workers received less than 50% of their very low salaries and sometimes only 1 out of 12 monthly salaries were paid. Widespread absenteeism, bribery and other corrupt practices attributed to frontline health providers are clearly unacceptable, but they need to be understood in the context of a dysfunctional state and governments, an underfinanced healthcare system, the plunder of rich natural resources and international financial flows towards tax havens.

## Funding and Ethics of Corruption Research

The paucity of good evidence about corruption interventions, especially from low- and middle-income countries should come as no surprise.^30^ The author searched the PubMed database with no time restriction for articles on corruption and health systems. In contrast to the search ‘Malaria and health systems’ which resulted in 2045 results (1968-2019) the ‘corruption and health systems’ search resulted in 98 results (1985-2019). Only 12 documents covered the complex issue linking political, grand and petty corruption. Why is this important?

The author worked from 1985 to 1990 as clinician and public health professional in Zimbabwe and observed the ‘Trickle down’ effect of corruption.^31^ My colleagues described it as ‘The fish rots from the head down.’ Significant petty corruption is only feasible in the presence of widespread grand and political corruption. In order to address the issues of systemic or institutionalized corruption and its important effect on the health system, more research on the relationship and interdependence between political, grand and petty corruption including effective interventions is required. The author is waiting to see funding provided for such research and the development of an international ethical approval mechanism to conduct such research on a scale appropriate to the size of the problem.

Unfortunately powerful actors with strong interests in the ‘status quo’ stand in the way of global research on systemic corruption. An opaque international financial sector which is not accountable to the global common good favours instead the global elite which is not interested in change. Therefore policy-makers and academics can only burn their careers, if they focus on such a ‘sensitive’ topic. Another important challenge is the need for an interdisciplinary, intersectoral and international study approach targeting national and international elites.

## Ethical issues

Not applicable.

## Competing interests

Author declares that he has no competing interests.

## Author’s contribution

RH is the single author of the paper.
